# Supplementary material for: Prevalence, causes, and factors associated with obstructed labour among mothers who gave birth at public health facilities in Mojo Town, Central Ethiopia, 2019: A cross-sectional study
Source: PLoS One. 2022 Sep 22;17(9):e0275170. doi: 10.1371/journal.pone.0275170 (PMC9499287; doi:10.1371/journal.pone.0275170)
Supplement: S1 File — (DOCX) [file pone.0275170.s001.docx]

**S1 File**. **English version questionnaire** **used to collect data on prevalence, causes, and factors associated with obstructed labour among mothers who gave birth at public health facilities in Mojo town, Mojo, Central Ethiopia, 2019**

| 101 | | Age? | | _______(in year) | | | |
| --- | --- | --- | --- | --- | --- | --- | --- |
| 1. 102 | | Residency? | | **1**)Urban  **2**)Rural | | | |
| 103 | | Educational status? | | **1**)illiterate  **2**)Primary  **3**)Secondary  **4**)Collage  **5**)Degree and above | | | |
| 104 | | Religion? | | 1)Orthodox  2)Muslim  **3)**Protestant  4)Catholic  5)Other | | | |
| 105 | | Did you have your own income? | | 1)Yes  2) no | | | |
| 106 | | If yes what your occupation? | | **1**)Private organization worker  **2)**Gov. employee  3)Merchant  4 private work  **5)** house wife  6)Other | | | |
| 107 | | Dose you have information on ANC and delivery service | | **1**)Yes  **2)** no | | | |
| 108 | | Distance from health facility by time? | | 1)0-30 min  **2)** 30(min)-1hr  3)1-2 hr  **4**) 2-3 hr  **5)**3-4  **6**) >4hr | | | |
|  | | **SECTION B: Obstetric history and other variables** | | | | | |
| 201 | | How many children you have? | | **1**)0  **2**)1  **3**)2  **4**)4  **5**)5 or more | | | |
| 202 | | Previous last birth with greater than 4 Kg ? | | | | **1**)Yes  **2)**No | |
| 203 | | Did you have ANC visit? | | **1)Yes**  **2)No** | | | |
| 204 | | If yes. Number of ANC visits? | | **1**) no visit  **2**)1 visit  **3**)2 visits  **4**)3 visits  **5)**4 or more | | | |
| 205 | | Where you follow-up? | | 1) Mojo hospital  **2**)from Other health facilities | | | |
| 206 | | If come from other facility referral means? | | 1)Referred by other facility  **2**)Self- referral | | | |
| 207 | | Diagnosis of mother at the hospital? | | 1)Normal labor  2)Malpresentation  3)Malposition  4)CPD  5)Placental abnormalities  6)Amniotic fluid abnormalities  7) IUFD  8)fetal heart beat abnormality  9) Prom  10)previous scar  11)other | | | |
| 208 | | Does partograph is properly filled? | | 1)complete  2) partially  3) not full field  4) other | | | |
| 209 | Total time of laboring? | 1. 0-12 hrs. 2. **2)** 13- 18 hrs. 3. **3**) 19- 24hrs 4. 4) 25-36hrs 5. **5**) > 36 hrs. | | | | |  |
| 210 | Mode of delivery? | | **1**)SVD)  **2)** Vacuum | | | **Surgical intervention**  **3)** (C/S) **4**) Laparotomy **5)**destructive delivery |  |
| 211 | Reason for procedure/intervention? If there is surgical intervention | | **1**)Fetal distress  **2**)Obstructed labor  **3**)previous scar  **4**)Malpresentation/ Malposition  **5**)Amniotic fluid abnormalities  **6**)APH/PPH  7) poor maternal contraction  8) other | | | |  |
| 212 | Previous abnormal/difficult in deliveries? | | **1)**Yes  **2)** No | | | |  |
| 213 | If yes? | | **1)** IUFD  **2)** big baby  **3)** Malpresentation/malposition  **4)** CPD  **5)** PROM  **6)** other**s** | | | |  |
| 214 | Did mother have complication now? | | **1)** Yes  **2)** No | | | |  |
| 215 | If yes Maternal complications? | | **1**)Sepsis  **2**)PPH  **3**)Ruptured uterus  **4**)Fistula  **5**)Death  **6)**Other | | | |  |
| 216 | Previous any chronic disease condition? | | ­­­­­­­­­­­­­­­­­­­­­­­­­­**1)**Yes  **2)**No | | | |  |
| 217 | Apgar score for the baby? | | | **1**)2-4  **2**)5-6  **3**) 7-8  d4>9 | | |  |
| 218 | Baby’s weight in Kilograms? | | **1**)<1.5  **2**)1.5-2.5  **3**)2.5-4  4)>4 | | | |  |
| 219 | Did a fetus have complication? | | **1**)Yes  **2**)no | | | |  |
| 220 | If yes Fetal/neonatal complication? | | **1**)Asphyxia  **2**)Death  3**)**Preterm  **4)** LBW  **5**) jaundice  6) other | | | |  |

**Finally, thank you very much for your cooperation**
Name and signature of Data collector ____________________________________________date____________
