# Supplementary material for: Prevalence, causes, and factors associated with obstructed labour among mothers who gave birth at public health facilities in Mojo Town, Central Ethiopia, 2019: A cross-sectional study
Source: PLoS One. 2022 Sep 22;17(9):e0275170. doi: 10.1371/journal.pone.0275170 (PMC9499287; doi:10.1371/journal.pone.0275170)
Supplement: S2 File — (DOCX) [file pone.0275170.s002.docx]

**S2 File.**  **Afan Oromo version of questionnaire used to collect data on prevalence, causes, and factors associated with obstructed labour among mothers who gave birth at public health facilities in Mojo town, Mojo, Central Ethiopia, 2019**

| 101 | Umurii hadhaa | | | _______(Bara) | |  |  |  |
| --- | --- | --- | --- | --- | --- | --- | --- | --- |
| 102 | Essaa jiraataa ? | | | 1)Magalaa  2**)**badiyyaaa | |  |  |  |
| 103 | Sadarkaa barnotaa ? | | | 1)Kaan barresussu dubbisuu hin dandenyee  2)Sadarkaa 1fffaa  3)Sadarkaa 2ffaa  4)kolleejjii  5**)**Digrii 1ffaa fi isaa oll | |  |  |  |
| 104 | Amantaa | | | 1)Orthodox  2)musilimaa  3)prootestaanntii  4)cathoolilaa  5)Kaan biraa | |  |  |  |
| 105 | Galii qabda (kaan ka”ee) | | | 1. Eyyeen 2. Laakii | |  |  |  |
| 106 | Yo galii mataa keetii qabatee dallaaggaa kee | | | 1)dhaabataa dhunfaa kessaa 5) Hadhaa waraa  2) Hoojeetaa mootummaaa 6) kaan biroo  3) dalddaala  4) hoojii dhunfaa | |  |  |  |
| 107 | Odeeffanoo wa’ee hordoofii ulfaa irratii qabaan? | | | 1)Qabuu  2)Hin qabaan | |  |  |  |
| 108 | Fageenyaa dhaabataa fayyaa irraa qabduu ? | | | 1) 0-30(daqiiqaa)4) Satii 2-3  2) 30(daqiiqaa) -1( Satii) 5) Satii 3-4  3)Satii 1-2 6)Satii > 4 | |  |  |  |
| **B** | **Kutaa B: halaa dahumsaa fi isaan kaan walqabatee** | | | | |  |  |  |
| 201 | daimmaan meqaa qabdaa ? | | 1)02)1  3)24)4  5)5 fi isa ol | | |  |  |  |
| 202 | Daimmaa kanaa duraa ulfatiinaa Kg 4 ol deessee ? | | 1)Lakii  2)eyyeen | | |  |  |  |
| 203 | Hordoofii ulfaa qabdaa? | | 1. Eyyeen 2. Lakkii | | |  |  |  |
| 204 | Hordoofii ulfaa yoo qabdaa tahee al meqaa demtee ilaalamtee | | 1. ala 0 2. ala 1 3. ala 2 4. ala 3 5. ala 4 fi isa ol | | |  |  |  |
| 205 | Eessaatii hordoofii ulfaa gochaa turtee | | 1. Hospitalaa mojo 2. Dhabaataa fayyaa biraa | | |  |  |  |
| 206 | Yoo Dhabaataa fayyaa biraa irraa dhuftee haalaa kamiin dhuftee | | 1. Referii 2. Ofiin | | |  |  |  |
| 207 | Dhibee argamee | | 1Ulfaa nagaa  2 Halaa malee dhufatii daimmaa  3)Halaa malee tahumsaa daimmaa  4)Ulfaa uuguramee  5)Ulfaa dheratee  6) Hanqinaa bishaaan daimmaa  7**)**dhibee dhigaa ulfaa waliin waal qabatee  8) daa”immaa garaa kessatii dadhabee  9) yeroo malee dhangaaluu fincaan daa”immaa  10) dahumsaa kanaa duraa baaqaaqsaanii yaluu  11) ulfaa yeroon dheratee  12) dhigaa dahuumsaan duraa  13) daimmaa guudaa  14) kaan biraa | | |  |  |  |
| 209 | | Halaan itii fayyadamaa waraqaa hordofiii cininsuu? | 1) Hin fayyadamnee  2)Murasaa fayyadamnii  3**)**Guutumaan guututii fayyadamaaniruu | | | |  |  |
| 210 | | Saatii meqaa cininsifatee ? | 1)0-12 saatii  2)13- 18 sataii  3)19- 24 saatii  4)25-36 saatii  5)> 36 saatii | | | |  |  |
| 211 | | Halaa dahuumsaa ? | | 1)Karaa gaddammeesaa  2) Afufeetin gotootuu  3)Opraasioonii | |  |  |  |
| 212 | | Yoo karaa gaddamessa hin tanee sababaa opraasionin hojetameefi ? | | 1**)**Afuraa dhabuu muccaa  2)Ulfaa uguramee  3) oprassionaan duraa  4)Halaa malee tahuu/dhufuu mucaa  5) daimaa ulfaatinaa gudaaa  6)dhibee dhangalaa mucaa waaliin wal qabatee  7 Dhigaa dahumsaa dura/bodaa  8)fashaalauu qorichaa mixuu  9) bora”uu dhangalaa dahumsaa  10) daa”immaa garaa kessatii dadhabee  11) dhibaa dhigaa  12) kaan biraa | |  |  |  |
| 213 | | Yoo Rrakkoo dahumsaa kanaa duraa turee ? | | 1. daa”immaa garaa kessatii dadhabee 2. daa”iimmaa akkumaa baheen dadhabee 3. ulfaa uguraamee 4. opraasiionii kanaa duraa 5. Halaa malee tahuufii /halaa malee dhufuu mucaa 6. Dhangala”uu bishaan ulfaa 7. Ulfaa bahee 8. Kaan biro | | |  |  |
| 214 | | Ulfaatinaa mucaa Kiloo graamidhaan? | | 1)<1.5  2)1.5-2.5  3)2.5-4  4)>4 | | |  |  |
| 215 | | Rakkoo hadhaa iraa gahee jiraaa | | 1. Eyeen 2. Lakkii | | |  |  |
| 216 | | Rakkoo hadhaa iraa gahee yoo jiratee ? | | 1)Kulkuluu qamaa  2)Dhangaluu dhigaa dahumsaa bodaa  3)Dhohuu gaddameessaa  4)Uraa walitii bahuu qamaa  5)Dua’aatii  6) kaan biraa | | |  |  |
| 217 | | Dhukkubotaa dhabatoo duraa qabdaa ? ­­­­­­­­­­­­­­­­­­ | 1)Jiraa  2)Hin jiruu | | | |  |  |
| 218 | | Maddalii Apgar | | 1)2-42)5-6  3)7-84)> 9 | | | |  |
| 219 | | Ulfaatinaa mucaa Kiloo graamidhaan? | 1)<1.5  2)1.5-2.5  3)2.5-4  4**)**>4 | | | |  |  |
| 220 | | Rakkoo dahumsaa kanaa duraa turee ? | 1)Jiraa  **2)**Hin jiruu | | | |  |  |
| 221 | | Rakkoo daa”mmaa iraa gahee jiraa ? | 1)Afuraa kutaa  2**)**Du’aatii mucaa yeroo dahumsaa  3) dahumsaa yeroo dursee  4) dua”aatii mucaa garaa hadhaa kessatii  5) mucaa xiqaa  6)bifaa kellon dhalachuu mucaa  7) garaa kessatii qancaruu mucaa  8**)**Kaan hin eraamnee | | | |  |  |

**Waan naa degertaaniif bayyee gallaatomaa**
maqaafi mallattoo gafii saassabaa ____________________________________________guyyaa

**S1 File: English version information and consent form**
**Information sheet**
My name is Tarekegn Girma I’m a Master’s degree student at Adama Hospital Medical College.
**Purpose** of the this study is to assess the magnitude, causes and associated factors of obstructed labor among women delivered at Mojo town

**Procedure:** The study involves a face-to-face interview with the data collector and observation at patient’s card. The data collector will ask a set of questions using a semi-structured questionnaire and review your card after signing the consent form, the Data collector will proceed to ask you the relevant questions and your responses will be recorded on the questionnaire. The interview will take about 5-10 minutes.
**Risk and benefits**: There are no known risks with the study. The results obtained may eventually aid decision makers and policy makers in reducing maternal mortality.
**Rights of Participants:** Your participation is voluntary. You are under no obligation; you may choose to participate or not to. If you decline not to participate, no privilege will be taken away from you. You can ask any question, which is not clear for you.
**Confidentiality**: any information you will give will be kept confidential, names will not be written or specified, and all the questionnaires will be coded for anonymity. Only the principal investigator will know the details and he will discard it after completing analysis. Are you willing to participate in this study? (1) Yes (2) No ________

**Informed Consent**; the Purpose of this study has been read to me in the language I comprehend and understand. The purpose, the benefits, risks, discomforts, and confidentially of the study has been explained to me. I further understand that: If I agree to take part in this study, I can withdraw at any time without having to give an explanation and that taking part in this study is purely voluntary; I agree to take part in this study. Sign:____________ Date____________

Data collector___________________Sign:____________ Date:___________
Researcher_____________________ Sign:____________ Date________

**S2 File: Afan Oromo version information and consent form**

**qorannichaa ibsuu** Akkam ! maqaan koo Tarraqany girmaan jedhama kollejjii fayyaa Adamaa irraayin dhufee
Kaayoon qorannoo kootii wa’ee ulfaa uguramaafi rakkollee inii ittin dhufuu.

**Hirmaattotni**: hadholee dahuumsaaf dhuufaanif
**Adeemsii isaa**: gaaffii fi deebiin gaafilee filannoo qabanii fi deebii keessaan itti dabaluuf banaa
ta’an of keesaa waan qabuuf yoo eeyyamammoo taataan gaaffii gaafatamtaniif deebii kennitanii ani immoo deebii keessaan baarreessa.Gaaffiin kunis daqiiqa 5-10 qofa fudhata.
**Miidhaa fi bu’a isaa**: midhaa tokkollee kan hin qabnee fi faayidaan isaa garuu duatii hadholee hirrisuu kessatii itti fayyaddamuu dandenyaa.
**Mirga Hirmaattota**: hirmaachuun keessan duursee fedhii irraatti hunda’a. namni isin dirqisisuu hin jiru. Yoo wanti isin hin tollee jiratee deebii kennuu dhiisuu ni dandeessuu,guutumattis adda kuttanii deemuu ni dandeessuu.
**Iccitiin isaaa eeggamadha**. Duursa maqaan keessaan hi bareeffamuu .deebii keessaan
qofatuu galma’a yoo isin eeyyamtaniin ala deebii sin kennitanis nama biroof hin kennamuu
nama qorannoo kana gaggeessu qofatuu itti fayadama. Yoo keessaatii hirmaatuuf eeyamammoo taatan **gaaffii koo ittii fufuu.** Deebiin isiin kennitaan
kan dhugaa irrattii hunda’ee qofa ta’uu qaba sababiin isaas sagantaa hirisuu duatii hadhollee keesatti itti fayyadamuun waan dana’amuuf. Qorannoon kun miseensota fudhatamummaa qorannoom mirkaneessaan kolejjii fayyaa hospitaalaAdaamaatiin kan mirkana’eedha.
itti fufuun ni danda’amaa? eeyyeen_____________ miti____________

Unkaa **eeyyamaammummaa**
Waa’een qorannoo kana sirrittii nattii himameera kaayyoo qorannoo kanaas hubadheera . rakkoo tokkollee jiruu kootii fi namummaa koo irrattii waan hin fidneef deegarsa tokko malee itti
hirmaadha. Sababaa ittii amaneef gaaffii qorannoo kanaaf deebii kennuuf eyyamamaa ta’uu koo mallattoo koon nan mirkaneessaa.

Hirmaata Mallatto barreeffaman ykn mallattoo quban_______________________

Maallattoo ogeessaa raga guuruu________________________

Maallattoo ogeessaa qorannoo gaggeessuu_________________________
