# Supplementary material for: Prevalence, causes, and factors associated with obstructed labour among mothers who gave birth at public health facilities in Mojo Town, Central Ethiopia, 2019: A cross-sectional study
Source: PLoS One. 2022 Sep 22;17(9):e0275170. doi: 10.1371/journal.pone.0275170 (PMC9499287; doi:10.1371/journal.pone.0275170)
Supplement: Attachment — Submitted filename: Response to reviewers.docx [file pone.0275170.s005.docx]

**Response to Reviewers**

Title: ***“Prevalence, Causes, and Factors associated with Obstructed Labour among Mothers who Gave Birth at Public Health Facilities in Mojo Town, Central Ethiopia, 2019”***

Manuscript ID: **PONE-D-21-26867**

Dear, Editor and reviewers, we (authors) would like to thank you for your time and fruitful suggestions. We respond and corrected all raised comments. Hereunder we enclosed all the answers and corrections point by point.

**Response to Editor’s comments**

1. Please ensure that your manuscript meets PLOS ONE's style requirements, including those for file naming.

**Authors’ response:** Dear Editor, Thanks a lot for your guidance with PLOS ONE's guidelines! We have corrected the entire manuscript as per PLOS ONE's requirements.

2. In your Data Availability statement, you have not specified where the minimal data set underlying the results described in your manuscript can be found.

**Authors’ response:** Great! We have updated the data availability statement and included all the data used in the study as supporting information in the revised manuscript.

3. We note that you have stated that you will provide repository information for your data at acceptance. Should your manuscript be accepted for publication, we will hold it until you provide the relevant accession numbers or DOIs necessary to access your data. If you wish to make changes to your Data Availability statement, please describe these changes in your cover letter and we will update your Data Availability statement to reflect the information you provide.

**Authors’ responses:** Dear, Thank you so much for your suggestion! We made changes to the data availability statement and described it in the cover latter.

4. Please ensure that you refer to Figure 1 in your text as, if accepted, production will need this reference to link the reader to the figure

**Authors’ response**: Dear Editor, Thanks for reminding us of our mistake. We have referred to figure 1 under the study variable sub-section of methodology.

5. Please include captions for your Supporting Information files at the end of your manuscript, and update any in-text citations to match accordingly.

**Authors’ response:** Thanks for reminding us! We have included supporting information and cited its corresponding captions in the text.

**Response to Reviewer #1 comments**

1. Needs English language correction.

**Authors’ response:** Dear reviewer, we are grateful for your suggestion. We have corrected all the language errors in our manuscript as highlighted in the track change file.

2. It is a good effort and may help in reduction of such complications

**Authors’ response**: Thanks a lot for your constructive comments!

**Response to reviewer #2 comments**

1. The area studied is of great clinical importance. Unfortunately, many women still experience labour complications and these contribute to the maternal morbidity and mortality

**Authors’ response:** Dear Reviewer, Thank you so much for acknowledging our study outcome (obstructed labour), one of the causes of maternal mortality and morbidity.

2. The manuscript is relevant; it has numerous grammatical and typographical errors that need to be corrected.

**Authors’ response:** Thank you for your fruitful comments!

We have corrected all the typographic and grammatical errors

3. The background/introduction has many sentences constituting paragraphs. Please coalesce most of them into relevant paragraphs and stratify them as follows:
   a) Important definitions such as Obstructed labour, including the burden of obstructed labour globally, in Africa/sub-Saharan Africa and in Ethiopia
   b) The causes of obstructed labour
   c) The factors associated with obstructed labour
   N.B: Not sure that the last sentence in the background about this being a pioneer study is relevant here. Which databases did the authors search to come to this conclusions?

**Authors’ response: Great!** We made significant changes to the introductory parts of our paper as per your suggestions. We stratified it as: definition and burden of OL, causes of OL factors associated, strategies that have been taken to prevent OL, and gaps in data about OL in the area. ***‘This is a pioneer study which assessed causes of obstructed 91 labour in the Central Ethiopia’*** just to express the limitation of evidence specifically, in our study setting. We have removed this section.

4. In the methods, the use of the term cephalo-pelvic disproportion is misleading. What exactly caused the disproportion is the real cause of obstructed labour. Please, if possible, unpack the cephalo-pelvic disproportion variable.

**Authors’ response:** OH GREAT! Really, it is insightful suggestion. Thanks! According to different literatures and guidelines CPD is taken as the first and leading cause of obstructed labour (as cited in the background section). In fact, there are root causes of CPD which are described as risk factor for obstructed labour such as maternal age, fetal macrosomia (hydrocephaly), contracted pelvis, etc.

5. Under birth weight, you write that a high birth weight is when the weight of baby at birth is 400gm and above. This is wrong. 400gm is too low to be a high fetal weight. I guess you meant 4000gm! Please correct this anomaly! The normal birth weight range is also not 2500gm-399gm but 2500-3999gm! Also correct this anomaly!

**Authors’ responses:** Dear, we are sorry for the mistake we made! It was a typing error! We meant 4000gm and 3999. We have corrected it. Thanks a lot for reminding us!

6. In the analysis part, please state how categorical and continuous variables were analysed instead of clamping everything together.

**Authors’ responses:** Excellent! Dear, the majority of the independent variables handled in our study were categorical in form, and before analysis, we also categorized continuous variables such age, labour time, and estimated distance to healthcare facilities.

7. In results, in Table 1 you state under occupation that the category "other" meant daily labour worker. Were there any other considerations under that category? If not, then just write daily labour worker directly in the table.

Under APGAR SCORE, what was the rationale of using the ranges the author uses? If possible, please stratify your APGAR SCORE as follows:

7–10: Reassuring

4–6: Moderately abnormal

0–3: Low

**Authors’ responses:** I really appreciate your helpful suggestion. Of course, there is another factor under the other category of Table 1 that was overlooked when typing, namely "students."

Dear, we have also changed the APGAR SCORE category in accordance with your recommendation.

8. Under discussion, the content is there but needs to be improved. Please write your discussion as follows: State your results, then results from comparable studies and the possible explanation for the similarities or differences between your study results and the quoted studies.

**Authors’ responses:** Dear reviewer, thanks for your guidance! We wrote the discussion section as per your recommendations!

**Response to reviewer #3 comments**

Major issues

1. Use of the word magnitude in the title is not specific enough, because this is very difficult to measure. The authors should consider revising this to prevalence

**Authors’ responses**: Dear Reviewer, We value your insightful suggestion. We have replaced the word "magnitude" with "prevalence" in the title section.

2. The introduction is very shallow and not informative because the authors have not reviewed and distilled the available literature on obstructed labour, yet it is a well-studied subject

**Authors’ responses**: Great! We made thorough revision to the introduction part and incorporated all relevant data to our study’s outcome interest. Thanks in advance!

3. Subsequently, the gap/problem as well as the justification is not clearly defined in the introduction.

**Authors’ responses**: Dear review, thank you for your concern over this important section! We have updated this section and we hope you will get the gaps and justification we articulated. Thanks!

4. The stated objective is not SMART.

**Authors’ responses**: Dear, thanks once more! We have corrected this section. We have corrected the study objectives and made them SMART.

5. Generally, the methods are poorly reported, the authors should make use of the STOBE checklist to improve on the transparency of reporting. It will also help to make it more comprehensive.

**Authors’ responses:** Dear, thank you in advance for your suggestions! We have filled and uploaded the STROBE checklist with supportive information.

6. Line 131- 132 is not clear, please check it and revise the grammar and sentence structure. Please do this for the rest of the document as well.

**Authors’ responses:** Dear, Thanks a lot! We have corrected the grammar and sentence structures of the mentioned section and in all the rest of the document.

7. The primary outcomes are two, but they are not well defined. It is important to report how, who and when the diagnosis of labour was made? Are there any guidelines for diagnosis of obstructed labour in your facility?

**Authors’ responses:** Dear reviewer, Thank you so much for your perceptive comments! The two outcomes of this study, Prevalence of obstructed labour and causes of obstructed labour were diagnosed by physicians. In Ethiopia, physicians use the Federal guideline for diagnosis and management of obstetrics cases.

(Federal Democratic Republic of Ethiopia Ministry of Health: Management Protocol on Selected obstetrics topics, January, 2010).

8. You did not include a section on study procedures. So, it is not clear when, how and who collected the data. When was the consent obtained? Did you review patient files or interview participants for data collection?

**Authors’ responses:** Great! Dear reviewer, we have described the study procedures in the ‘data collection tools and procedure’ sub-section of methods and material.

- The data were collected by two BSc Midwives (who works out of the study area) and a supervisor from 10 November to 30 December 2019.
- Consent was taken from each participant before interview.
- Great! We have used both interview and patient file review.
- After data collectors taken consent, they interviewed study participants and reviewed the respective clinical information from medical record.

9. The results are not well presented. For instance, in Table 2, why do you have three variables on the same factor of ANC? What are they showing? Generally, there is a big problem with the categorizations on several variables; it is so arbitrary and unconventional. In table 3, under partograph is another example of the same.

**Authors’ responses:** Dear reviewer, Thank you so much for your insightful comments! Dear, we have unpacked the category of having ANC information and left having ANC follow-up and frequency of ANC follow-up. Because, evidence shows that frequency of ANC has association with obstructed labour. Mostly, those mothers who had less ANC follow-up were risky for OL. We have corrected all of the commented sections.

10. Table 4 is presenting outcomes of obstructed labour; this is not one of the objectives for this study. This is completely new and not acceptable.

**Authors’ responses:** Dear, we appreciate you for your intellectual suggestion!

Of course, outcome of obstructed labour was not our study objective but we included the feto-maternal outcome of participants in the study area as an explanatory variable. Because, as literatures shown, feto-maternal outcomes such as birth weight affects labour outcomes. Dear, to make it more clear and attractive for readers, we have merged the important feto-maternal outcome variables to the obstetrics characterises section of the findings.

11. In the discussion, it is hard to know what the key message is. This has also affected the conclusions and recommendations that have been advanced by the authors.	Authors’ responses: Dear, thanks for your helpful suggestion! We revised the discussion section. We concluded what we found and forwarded as per our findings. Thanks in advance again!

**Minor issues**

1. Kindly pay attention to the grammatical errors and typos that are all over the document	**Authors’ responses:** Dear reviewer, thanks for your constructive comments! We have corrected all the grammatical errors and typos throughout the document.
2. Have a look at the references as well and ensure that they are more accurate.

**Authors’ responses:** Thank you! We have checked and corrected it.

3. Many sentences are either incomplete or not meaningful.

**Authors’ responses:** Thank you dear! We have checked and corrected all the sentence errors all over the manuscript.

4. The paragraphs are not well structured, it is not proper to have one sentence standing alone as a paragraph.

**Authors’ responses:** We greatly appreciate you! All of the paragraphs have been rearranged, and we have fixed all the mentioned errors.
