# Supplementary material for: Prevalence, causes, and factors associated with obstructed labour among mothers who gave birth at public health facilities in Mojo Town, Central Ethiopia, 2019: A cross-sectional study
Source: PLoS One. 2022 Sep 22;17(9):e0275170. doi: 10.1371/journal.pone.0275170 (PMC9499287; doi:10.1371/journal.pone.0275170)
Supplement: Attachment — Submitted filename: Response to Reviewers.doc [file pone.0275170.s006.doc]

**Response to reviewers**

**PONE-D-21-26867R1**

**Prevalence, causes, and factors associated with obstructed labour among mothers who gave birth at public health facilities in Mojo Town, Central Ethiopia, 2019: A cross-sectional study**

Dear Dr. Gulzhanat Aimagambetova (Editor) and reviewers, we appreciate your valuable time with our article. Thanks for your intellectual comments and suggestions. We have revised our article and responded point-by-point to all the editor and reviewers comments and suggestions hereunder.

ACADEMIC EDITOR:

The introduction part sounds clear and provides the study rationale well. However, I suggest rewriting the last paragraph (lines 95-101 in the revised document). The issue is that you write "the aim of this study was" (singular), however below you stated THREE aims (plural). So, this should sound more clear. Moreover, the writing style should be improved in this paragraph to be closer to the academic writing style. It is better to remove the numbering (1,2,3) and present it in plain text.

**Authors’ response:** Dear Editor, Thank you so much for your intelligent suggestion. We have changed ‘aim’ to ‘aims’ and ‘was’ to ‘were’ in the mentioned section. Also, we have removed the numbering from the objectives of the study and presented it in plain text (highlighted on page no. 5; line no. 92-94).

In the Methods part, you described "Operational definition" (singular), but in the text there are many definitions, so the subheading should be "Operational definitions".

**Authors’ response:** Dear Editor, Thanks once again. We have changed "Operational definition" to be "Operational definitions"(highlighted on page no. 7 and line no. 141).

Please make sure you've followed the PLOS ONE journal's requirements

**Authors’ response:** Dear Editor, we have followed the PLOS ONE journal's requirements.

**Review Comments to the Author**

**Reviewer #1:** Overall a good effort in writing the manuscript, however the comparison with other studies should have been presented in a standard way.

**Authors’ response:** Dear reviewer, thank you so much for your concern on this important section. We have corrected the mentioned section (highlighted on Page number 17; line number 290 and 291).

Recommendation must mention the strategies to prevent obstructed labour in hospital settings such as training of birth attendants to monitor and identify cases who may land up in obstructed labour.

**Authors’ response:** Dear reviewer, thank you so much for your valuable suggestions. We have mentioned the strategies to prevent obstructed labour in hospital settings as per your suggestion (highlighted on page number 17; line number 295 and 296).

**Reviewer #2:** The author has addressed the comments raised by the reviewers. The manuscript reads much better than before.

**Authors’ response:** Dear reviewer, thank you for your time with article.

**Journal Requirements:**
Please review your reference list to ensure that it is complete and correct. If you have cited papers that have been retracted, please include the rationale for doing so in the manuscript text, or remove these references and replace them with relevant current references. Any changes to the reference list should be mentioned in the rebuttal letter that accompanies your revised manuscript. If you need to cite a retracted article, indicate the article’s retracted status in the References list and also include a citation and full reference for the retraction notice.

**Authors’ response:** Dear, we have revised the reference lists and completed and corrected some of missed contents of the references such as sure name, page number, DOI, Journals, and publishers. Additionally, we have replaced Reference no. 41 by a new relevant reference. The previous reference was incorrect and unintentionally placed during automated citing by Mendeley library.

**Thanks all for your time!**
